# Lipidomic Trajectories Characterize Delayed Mucosal Wound Healing in Quiescent Ulcerative Colitis and Identify Potential Novel Therapeutic Targets

**DOI:** 10.7150/ijbs.67112

**Published:** 2022-02-14

**Authors:** Jacob Tveiten Bjerrum, Yulan Wang, Jingtao Zhang, Lene Buhl Riis, Ole Haagen Nielsen, Jakob Benedict Seidelin

**Affiliations:** 1 Department of Gastroenterology, Medical Section, Herlev Hospital, University of Copenhagen, Denmark.; 2 Singapore Phenome Centre, Lee Kong Chian School of Medicine, Nanyang Technological University, Singapore.; 3 Department of Pathology, Herlev Hospital, University of Copenhagen, Denmark.

**Keywords:** Eicosanoids, inflammatory bowel disease, metabolomics, metabonomics, mucosal healing, phospholipids

## Abstract

Improving the long-term prognosis of ulcerative colitis (UC) requires sustained deep mucosal colonic healing with histologic remission, making the study of colonic tissue regeneration essential. In experimental colitis models, lipid metabolites are recognized as pivotal components of this process. This study aimed to describe the kinetics of wound healing and lipid metabolites engaged in regeneration in the normal colonic mucosa and how they are affected in UC to reveal new therapeutic targets. Experimental colonic wounds were created endoscopically in quiescent UC (n=21) and controls (n=9), and the healing process was surveilled by serial endoscopies and cross-sectional wound biopsies post-wounding. Biopsies were analyzed by liquid chromatography coupled with mass spectrometry. Endoscopic wound scores were significantly higher in UC at day two (p=0.001) and seven (p<0.0001) post-wounding, demonstrating a prolonged wound healing process. The wound scores were correlated with lipid mediators crucial for normal regeneration and sustained UC-specific changes in key phospholipids and eicosanoids, i.e., lysophosphatidylcholine, phosphatidylcholine, lysophosphatidic acid, phosphatidylglycerol, phosphatidylinositol, prostaglandin D_2_, and prostaglandin E_1_, were observed. A prolonged wound healing process is identified in quiescent UC with altered disease specific lipidomic trajectories providing potential novel therapeutic avenues for stimulating mucosal regeneration as an add-on to the traditional immune suppression treatment.

## Introduction

Ulcerative colitis (UC) 1 is the most prevailing entity under the umbrella term of inflammatory bowel disease (IBD) with an increased incidence and prevalence worldwide, although a plateau seems to have been reached in the Western world 2. The disease is characterized by inflammation and concomitant wounds in the colon. Affected individuals experience a substantial impact on the quality of life during phases of flares, characterized by bloody diarrhea and abdominal discomfort 3. UC carries a cumulative risk of colectomy of 7.5% after 5 years 4, and certain subgroups (e.g., concomitant primary sclerosing cholangitis, early onset, extensive and long-standing activity, or a familial history of colorectal cancer (CRC)) have a slightly increased risk for development of CRC 5. In recent years it has gradually become evident that the main risk factors for disease activity, colectomy, or CRC are sustained wound formation and inflammation, and that medication improving mucosal healing modifies the disease course favorably 6. On the other hand, there are several lines of evidence showing that anti-inflammatory strategies might impair important pathways involved in mucosal tissue regeneration 7. Accordingly, research on wound healing in the healthy colon and in patients with UC is critical in order to identify potential therapeutic targets that could modify the disease course through promotion of tissue regeneration.

Research within the lipidome - a subset of the metabolome - is receiving increased attention 8 as it represents lipid metabolites that are ubiquitous and with a surprising complex plethora of bioactive functions 9. One such member of the lipidome, prostaglandin E_2_ (PGE_2_), a principal eicosanoid with multiple functions, has been shown to promote early wound repair in mice by inducing wound-associated epithelial (WAE) cells, forming an initial barrier over the wound 10. Once a wound is closed by WAE cells, the level of PGE_2_ diminish, which is essential for the sequential wound healing process, as a continuously high level of PGE_2_ inhibits fibroblast migration, represses normal stem cell functions, and drives cells into a constant proinflammatory state as seen in vitro as well as in murine models of colitis 10-12. After PGE_2_ levels subside during wound repair, the anti-inflammatory PGD_2_ and its metabolites rise, and the wound healing process enters the resolution phase 13. Here resolvin E1, another eicosanoid and an endogenous lipid mediator derived from omega-3-fatty acids, inhibits the inflammatory process and promotes epithelial cell migration and proliferation, and ultimately wound repair in an experimental mouse model of mucosal wound healing 14. Another key lipid is lysophosphatidic acid (LPA), which is an intermediate phospholipid released by activated platelets and fibroblasts upon injury. This metabolite promotes platelet aggregation and induces cellular tension and cell surface fibronectin assembly, which are key events in the wound healing process 15. Thus, eicosanoids and phospholipids are considered pivotal components in the various stages of the wound healing process, and studies in both human 16 and experimental colitis models 14,17 have provided promising treatment results with some of these lipid metabolites. However, only very few of the lipids are well-described in human wound healing, and due to the lack of applicable human *in vivo* models, research on the lipidome and human colonic mucosal wound healing remains unexploited.

In this study, a novel human *in vivo* colonic wound healing assay was applied in which small experimental wounds were created with a biopsy forceps in the human colonic mucosa. The wound healing process was surveilled by serial endoscopies at day two and seven post-wounding during which a wound score was calculated and biopsies across the experimental injuries were obtained. Multiple initial injuries were made in order to sample only one wound healing biopsy from each injury site. Multiple sampling from the same injury site was thus not performed. The serial biopsies were subsequently analyzed by liquid chromatography coupled with mass spectrometry (LC-MS) producing a metabolic profile focusing on phospholipids and eicosanoids. Using this approach, the aims were: 1) to describe the kinetics of colonic mucosal wound healing in healthy individuals and patients with quiescent UC, 2) to characterize the lipid changes during wound healing, and thus 3) to identify the most likely new potential therapeutic targets that might be manipulated pharmacologically to enhance tissue regeneration among patients with UC.

## Materials and methods

### Population

Cohort 1, a test cohort for the initial histological evaluation of the wound healing process. Eight patients with UC in remission and eight healthy controls were included. The results were used to provide a rational timeframe for the observation points in the subsequent Cohort 2.

Cohort 2, the kinetics and lipidomics cohort. Sample size calculation is an inherent problem in omics analyses, however, based on our previous extensive experience with metabonomics we estimated that eight patients in each group were the absolute minimum to detect differences of relevance 18-20. Thus, twenty-one patients with quiescent UC as well as nine healthy control subjects were included.

All patients were recruited from the outpatient clinic at the Dept. of Gastroenterology, Herlev Hospital, University of Copenhagen, Denmark, and only patients with an affirmed diagnosis of UC according to well-established criteria 1 were included. The disease had to be in complete endoscopically (endoscopic Mayo sub-score 0) 21, histological (Geboes score < 0.1) 22 and clinically remission (total Mayo score < 2) 21 at time of inclusion. On-going maintenance treatment with 5-aminosalicylic acid, thiopurine, and biologics was allowed but needed to be on a stable dosing at least 3 months prior to inclusion. The healthy controls were volunteer subjects without any known diseases and free of daily medication. No dietary restrictions were imposed.

All participants included were between 18 and 75 years old, without a history of cancer, and without any other medication affecting the immune system apart from the above-mentioned stable maintenance medication. Lactating or pregnant subjects as well as subjects with psychiatric or neurological disorders that would affect decision-making were excluded from participating in the study.

The project, including the *in vivo* wound assay on humans, was approved by the Scientific Ethics Committee of the Copenhagen Capital Region (Protocol No. H-18003763).

### The human *in vivo* wound healing assay

**Figure 1:**
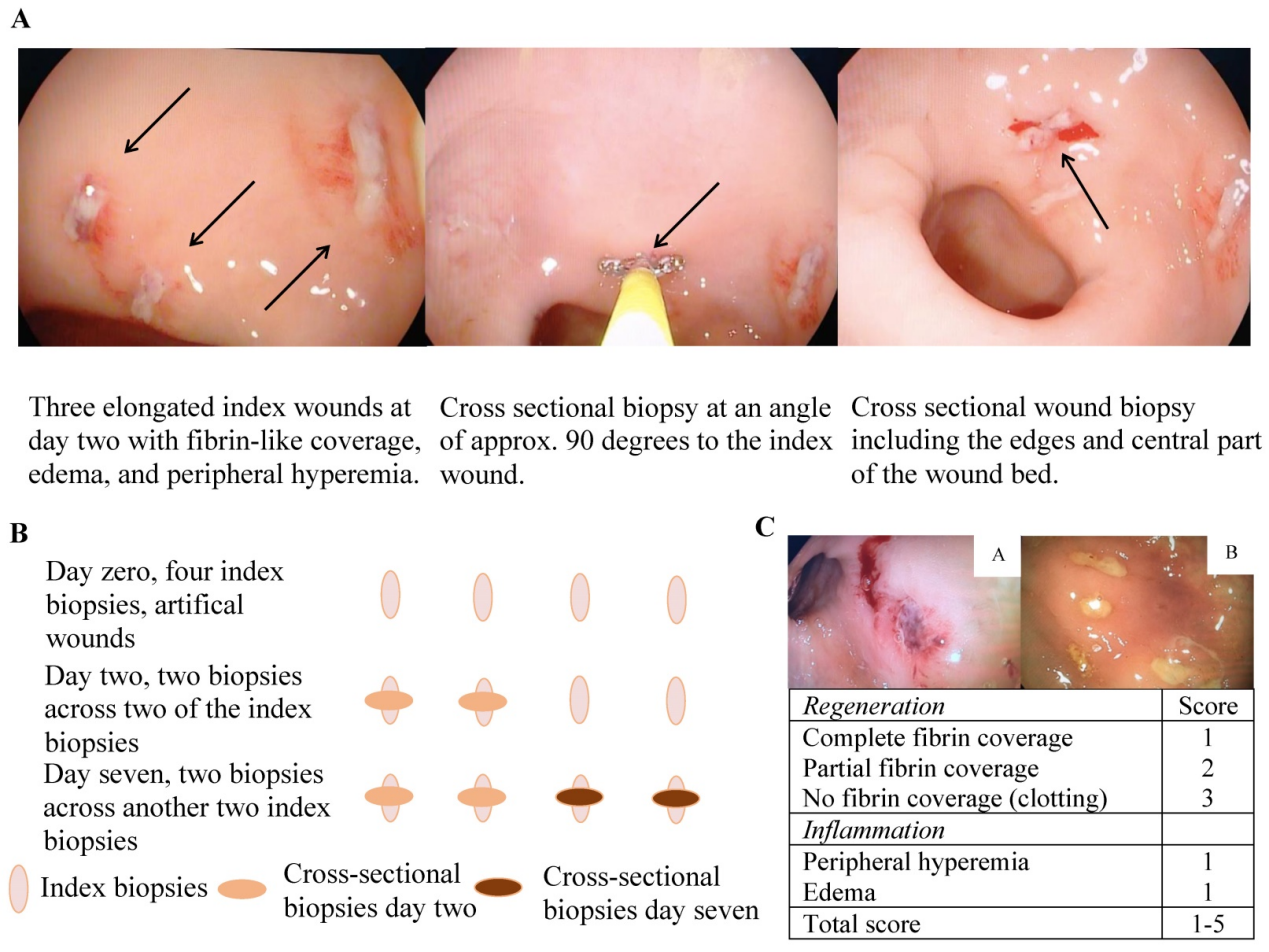
** The wound healing model.** A and B) illustrative presentation of the model. C) The colonic mucosal composite wound healing score ranges from 1-5 with two variables: 1) a regeneration score ranging from 1-3 and 2) an inflammation score of 1 or 2 - a high wound score correlates with impaired wound healing, e.g. a, wound score 5 with no fibrin coverage (3 points), marked peripheral hyperemia (1 point) and edema (1 point), thus representing delayed wound healing, and b; wound score 2 with complete fibrin coverage (1 point) and edema (1 point) representing timely wound healing.

A simple human *in vivo* colonic mucosal wound healing model (Figure 1A and B) and concomitant wound scores (Figure 1C) were applied as previously described 23. In short, an experimental wound was generated in the rectosigmoid part of the colon by use of biopsy forceps (Radial Jaw^tm^ 4, 2.8 mm, Boston Scientific, Marlborough, MA, USA), which generated an elongated wound of approximately 7 × 2 mm and extended into the lamina muscularis mucosa only. To avoid unnecessary mechanical forces affecting the wound healing process and intersegmental variation, the wounds were placed in between mucosal folds at the rectosigmoid intersection. The wound and healing processes were documented by successive macroscopic high-definition imaging and recorded through a standard endoscope at the given time points, and all digitally recorded procedures were independently reviewed and scored by two gastroenterologists in a blinded fashion. Subsequent molecular characterization was made possible by obtaining biopsies across the experimental wounds at the pre-defined time points with the use of a biopsy forceps angled 90 degrees to the initial experimental wounds. Importantly, each experimental wound was sampled only once to avoid repeated sampling across a single wound. The model consequently allowed a description of the kinetics of wound healing in the human colonic mucosa on both the macroscopic and molecular levels.

Participants in Cohort 1 had a sigmoidoscopy performed at day zero, one, and two, whereas subjects in Cohort 2 had a sigmoidoscopy at day zero, two, and seven. Bowel preparation with enemas was performed prior to each sigmoidoscopy. At the index endoscopy, four biopsies were obtained; three biopsies were snap frozen and stored at -80 °C, and one biopsy fixed in formalin. At the two subsequent sigmoidoscopies, two biopsies were obtained exactly across the index biopsies (Figure 1B); the first biopsy was snap frozen and stored at -80 °C and the second fixed in formalin.

### Histological assessment of inflammation

Inflammation was assessed using the Geboes score 22, which grades inflammation and structural changes associated with UC on a scale from 0.0 - 5.4. However, a modified Geboes score was applied leaving out grade of “ulceration”, as all of the samples had ulcers from the experimental wound. The modified Geboes score thus ranged from 0.0-4.4.

### Lipidomics

#### Sample preparation

The tissue samples were weighed into their respective bead beating tubes. A total of 50 µL of phospholipid internal standard mixture, 5 µL of oxylipins internal standard, 10 µL of butylated hydroxytoluene (BHT) and 435 µL of methanol were added into the respective tubes 24. Tissue samples were homogenized and incubated at 4 ^º^C for 1 hour. Samples were then centrifuged for 10 min at 10,000 *g* and 4 ^º^C. Five µL of the supernatant was diluted 500 times for phospholipid quantification in positive ion mode, and 350 µL of the supernatant was dried and reconstituted in 50 µL of methanol for quantification of phospholipid and eicosanoids in negative ion mode. Two µL of the reconstituted sample was pooled for phospholipid identification in negative ion mode, and a 100 times dilution of the pooled sample was used for phospholipid identification in positive ion mode.

#### Ultraperformance liquid chromatography-mass spectrometry (UPLC-MS) qualification of phospholipids and eicosanoids

An ACQUITY UPLC system coupled tandem quadrupole mass spectrometer (Xevo TQ-S, Waters, Milford, MA, USA) operated in electrospray ionization (ESI) mode and reverse phase UPLC column, ACQUITY UPLC BEH C18 column (2.1 mm × 100 mm, 1.7 µm, Waters), was employed for the separation and detection of these compounds. The data acquisition was performed in multiple reaction-monitoring (MRM) mode. For phospholipids, capillary voltage was set at 3.20 kV and 2.00 kV for the positive and negative ion mode runs, respectively. The phospholipids were separated on an isocratic flow of 0.4 mL/min for 6 min with A (methanol (98%), water (2%), ammonium acetate (5 mM), and formic acid (0.01%)) for positive detection, or 5 min with B (methanol (98%), water (2%), ammonium acetate (10 mM), and formic acid (0.01%)) for negative ion mode detection. The column temperature was maintained at 45^ º^C, and the injection volume was 2 µL for both ion modes. The separation of eicosanoids were performed at a flow rate of 0.6 mL/min with an initial gradient of 25% B (isopropyl alcohol:acetonitrile (90:10)) and 75% A (water with formic acid (0.1%)) for 1 min, then a linear gradient to 95% B at 8 min, held until 8.5 min. The total injection volume was 5 µL, and the column temperature was maintained at 40 ^º^C. The capillary voltage was set at 2.5 kV, source temperature at 150 ^º^C, and a desolvation gas flow rate of 1000 l/hour at 600 ^º^C.

For internal standards, chemicals, and quantification analysis please see Supplementary material.

### Data analysis

#### Univariate analysis

All continuous variables, i.e., wound score, Geboes score, biopsy weight, and lipids, were analyzed with nonparametric statistics using GraphPad Prism (v9.0.0, GraphPad, San Diego, CA, USA) and data presented as medians with interquartile ranges. The Mann-Whitney and Wilcoxon tests were applied when relevant with a significant level of p<0.05. A false discovery rate (FDR) below 5% was used for correction of multiple testing.

#### Multivariate data analysis

Multivariate statistical analyses were initiated with principal component analysis (PCA) to visualize the general structure of the mean centered data set and to identify any abnormalities or outliers (based on the principals of Hotelling´s T^2^) using SIMCA-P+ (v12.0, Umetrics, Umeå, Sweden). Class-belongings, i.e., control or UC day zero, two, or seven, and variables, were characterized by supervised models using the orthogonal partial least squares discriminant analysis (OPLS-DA) on data scaled to unit variance (UV). The models were validated with a seven-fold cross validation and permutation tests (200 permutations). The significance of the OPLS-DA models was additionally validated by the analysis of variance of the cross-validated residuals (CV-ANOVA), and a p-value < 0.05 indicated a valid model. The models were considered valid only if the permutation test and the CV-ANOVA test were satisfied at the same time. Variables holding strong differential power in the models were identified with the use of correlation coefficients. Similarly, correlation analyses and validations were performed between the colonic mucosal wound scores and phospholipids and eicosanoids, respectively. All data are deposited at MetaboLights.

## Results

### Clinical characteristics and samples

Clinical data for both cohorts are detailed in Table 1. All samples from day zero were obtained without any signs of histological inflammation; Geboes score < 0.1 22, confirming the clinical and endoscopic Mayo scores 21, and thus, ensuring deep mucosal healing in patients with UC.

On average, samples had a wet weight of 4.89 mg, however, significant differences were found between the average weight of index biopsies and the subsequent cross-sectional wound biopsies (Figure S1).

### Cohort 1, Modified Geboes scores

**Figure 2:**
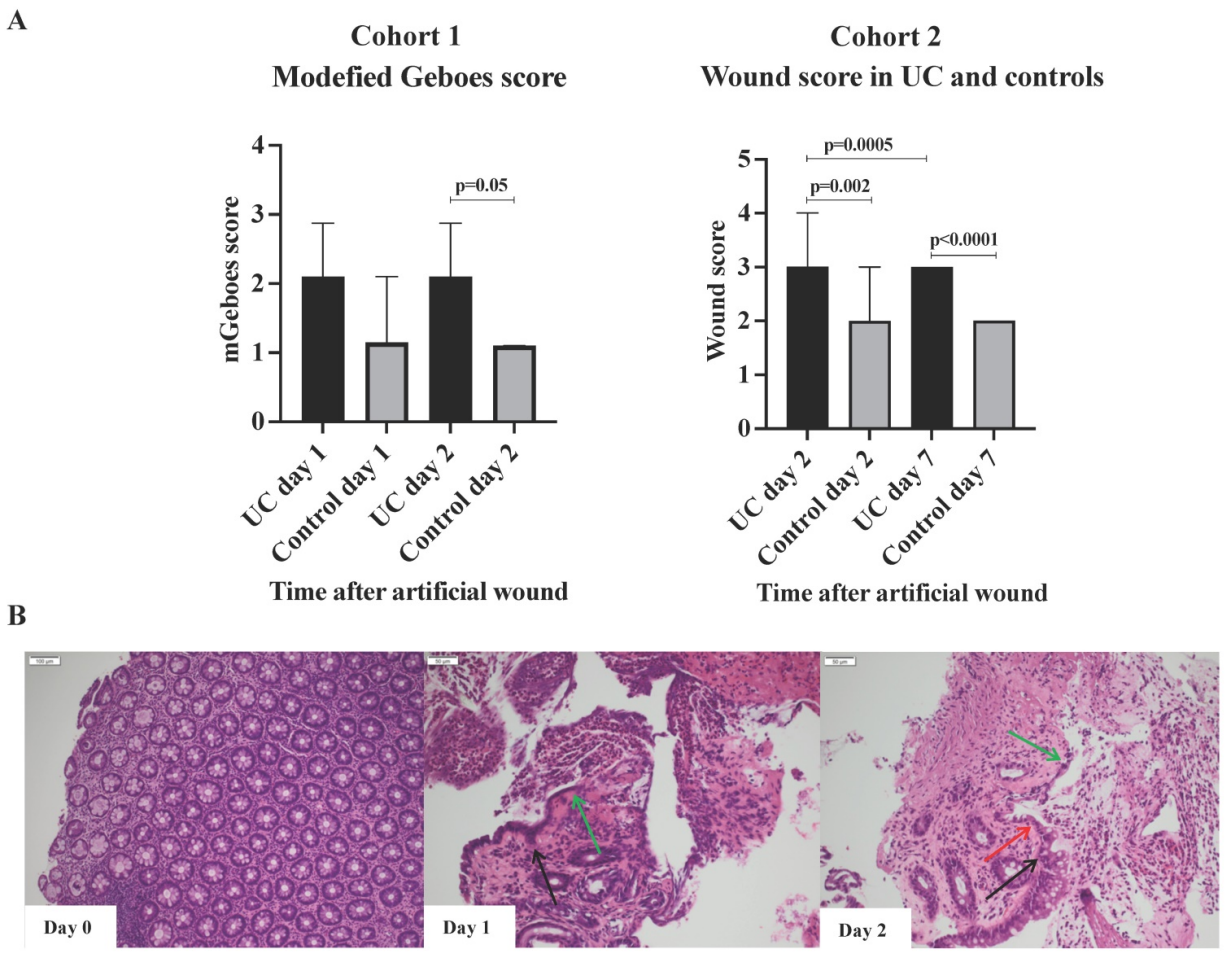
** Wounds and histology.** A) Modified Geboes and wound scores, median and interquartile range. The majority of subjects had a wound score of 3 (UC) or 2 (Control) at day 7 explaining the lack of range. B) Illustrative example of the histological appearance of the injury with acute inflammation. Black arrows, the edges of epithelial cells; green arrows, cuboidal regenerative epithelial cells; red arrows, flattened elongated wound associated epithelial (WAEs) cells. WAEs first appear after 48 hours.

No difference in the modified Geboes scores was observed within each group when day one and day two were compared (Figure 2A). However, when comparing the two groups at day one and two, a clear trend was found with higher modified Geboes scores (at day 2, 2.2 *vs.* 1.1; p = 0.05) among patients with UC indicating a more intense inflammatory response to wounding. The histological evaluation also revealed that cells resembling WAEs did not appear until 48h post-wounding in both UC and controls (Figure 2B). These observations indicate that the early inflammatory response to injury might be more rigorous and sustained in patients with UC as previously described 23, and that the regenerative healing process extends beyond day two. Thus, to ensure that the actual wound healing process was investigated, and not just the inflammatory response caused by the wounding, subjects in the subsequent cohort 2 had sigmoidoscopies performed at day zero, two, and seven.

### Cohort 2, Wound scores

The wound scores at day two after the artificial wounding were significantly higher in UC than controls (3 *vs.* 2; p=0.002), primarily due to insufficient fibrin-like coverage and peripheral hyperemia (Figure 2B). The significant difference in wound scores between UC and controls persisted at day seven (p<0.0001). At day seven, all patients with UC had achieved complete white fibrin-like coverage, but peripheral hyperemia and edema were, however, on-going characteristics.

### Lipidomics data

#### Univariate analysis

**Figure 3:**
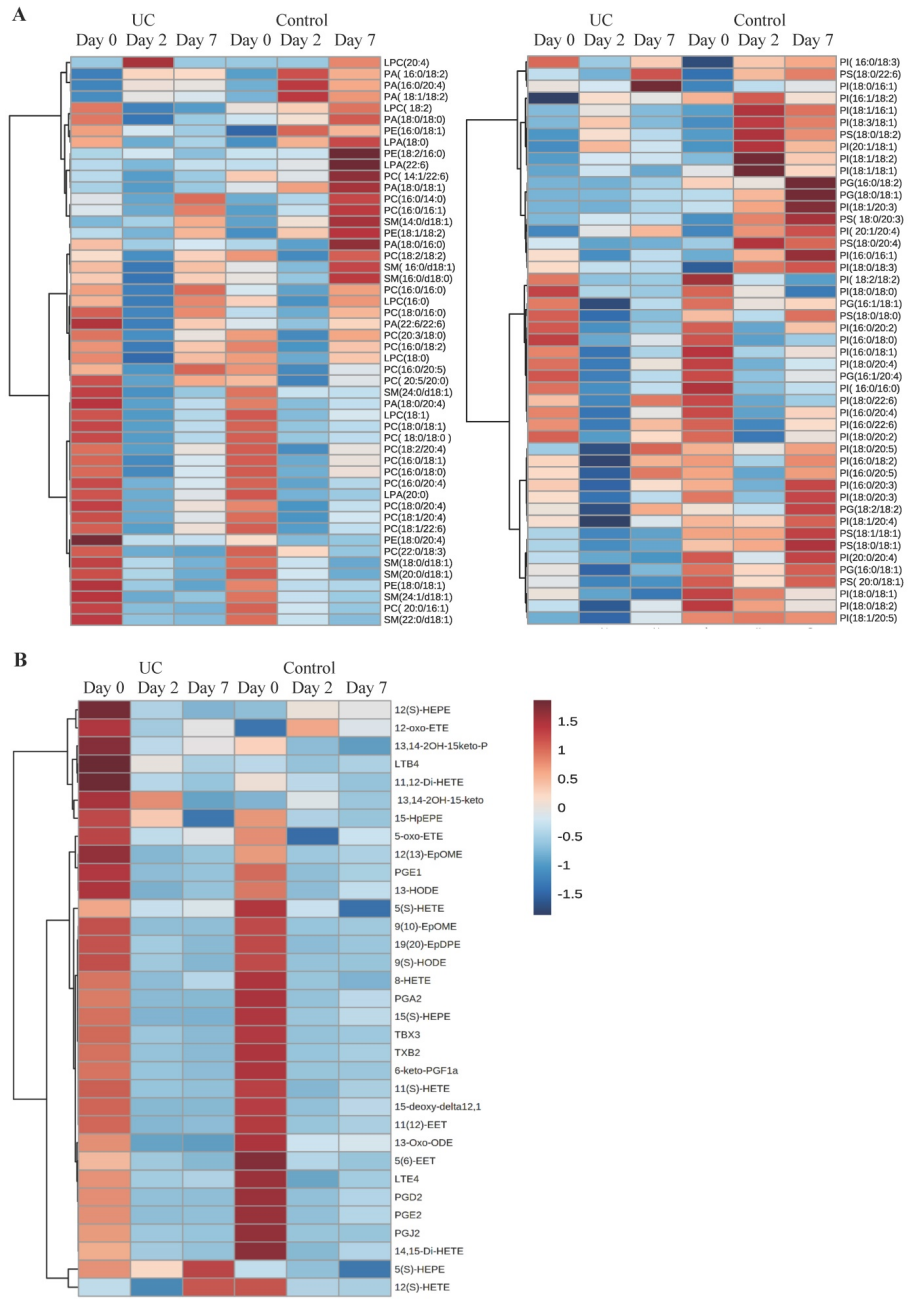
**Heat maps of phospholipids and eicosanoids.** Columns represent ulcerative colitis (UC) and healthy controls at day 0, 2, and 7, respectively. Rows represent each measured A) phospholipid or B) eicosanoid. Red and blue indicates relative increased or decreased concentrations.

**Figure 4:**
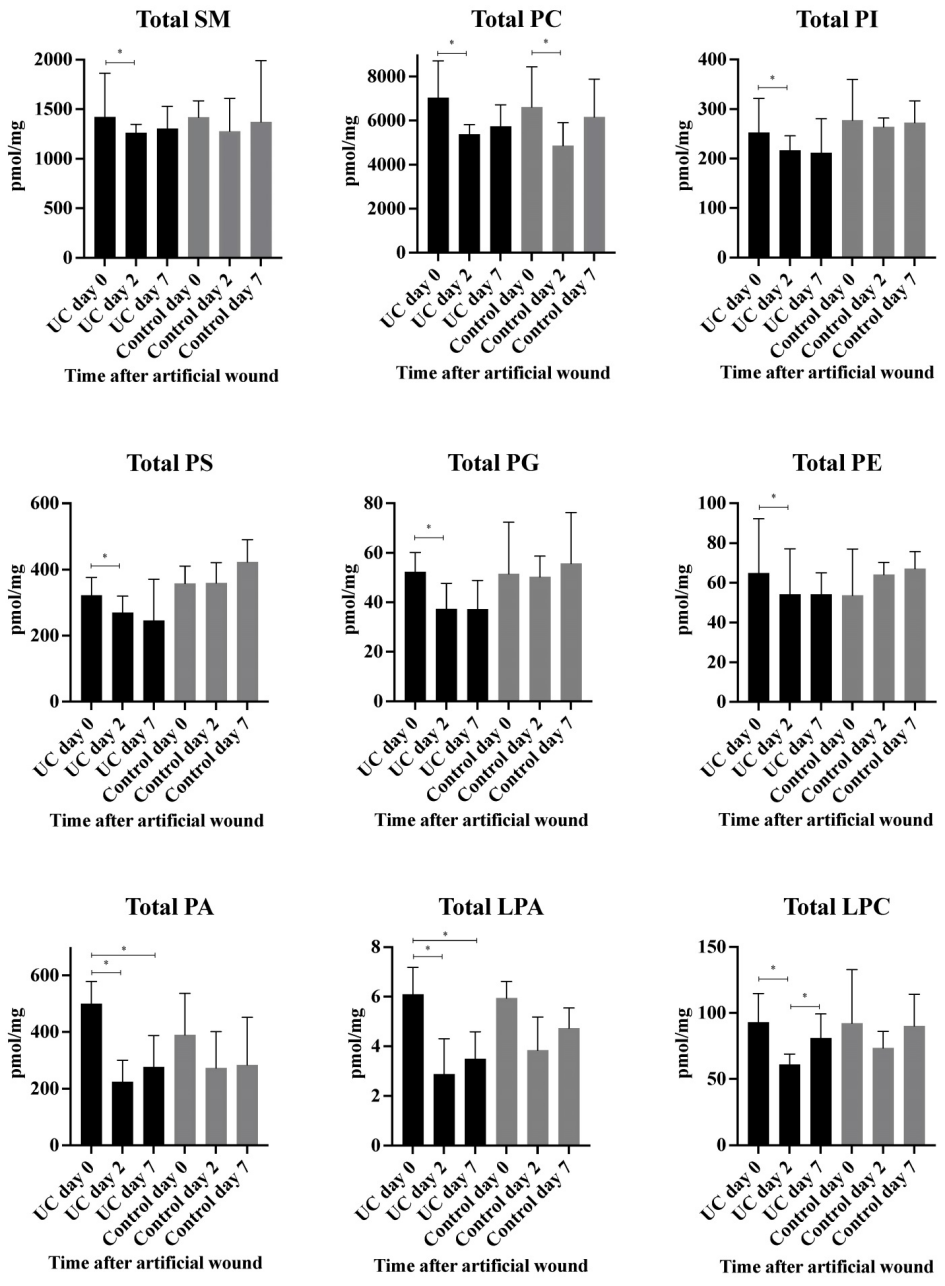
** Measured total concentration of phospholipids (pmol/mg) during wound healing in ulcerative colitis and controls.** Median and interquartile range. * Significant with a False Discovery Rate p<0.05. Lysophosphatidic acid (LPA), lysophosphatidylcholine (LPC), phosphatidic acid (PA), phosphatidylcholine (PC), phosphatidylethanolamine (PE), phosphatidylglycerol (PG), phosphatidylinositol (PI), phosphatidylserine (PS), sphingomyelin (SM), ulcerative colitis (UC).

A total of 98 phospholipid structures were detected and identified (Table S1). Comparing UC and controls, a significant lower amount of (16:0/18:2) phosphatidylglycerol (PG) was revealed at day seven. Further, a striking pattern was observed in the heatmap (Figure 3A) with significant differences within all examined classes of phospholipids at day two and seven as compared with day zero for UC. In contrast, only few and transient changes were observed in controls with no significant differences between day zero and seven. This pattern became especially evident when looking at the total amount of the different phospholipids measured (Figure 4) in which significant (p<0.05) changes were observed at day two within all phospholipid classes in UC. These changes were sustained at day seven, although only at a significant level for phosphatidic acid (PA) and LPA. The control group, on the other hand, only had a significant and transient reduction in phosphatidylcholine (PC).

A total of 33 eicosanoids were detected and identified (Table S2). Prominent and significant (p<0.05) reductions were observed within each group at day two and seven when compared to day zero, of which some were specific for UC, i.e., PGE_1_, 13,14-2OH-15keto-PGD_2_, PGJ_2_, 5(S)-hydroxyeicosapentanenoic acid (5(S)-HEPE), 13-hydroxyoctadecadienoic acid (13-HODE), and 14,15-dihydroxyeicosatetraenoic acid (14,15-di-HETE), whereas other lipid metabolites were specific for controls, i.e., 5(S)-HETE, 8-HETE, and 5(6)-epoxyeicosatrienoic acids (5(6)-EET). Nonetheless, no differences were noted when comparing UC and controls at the three time points (Figure 3B).

#### Multivariate analysis

PCA score plots from phospholipid and eicosanoid profiles containing all 90 samples identified five outliers. All five participants, from which these outliers originated, were reviewed in respect to demographics, clinical, and paraclinical parameters, including lipidomic data, but no obvious explanation could be identified, and the samples were consequently included in subsequent analyses.

**Figure 5:**
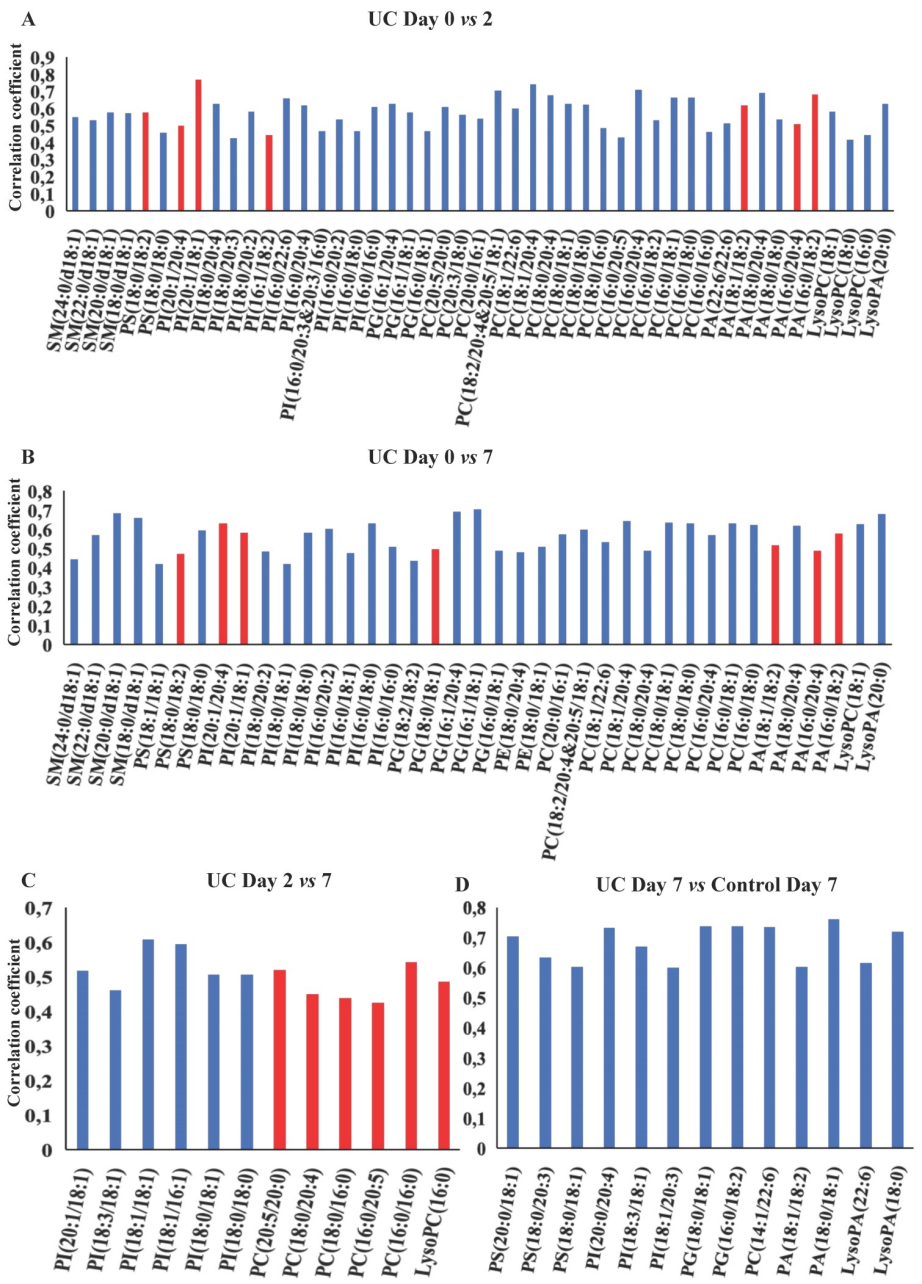

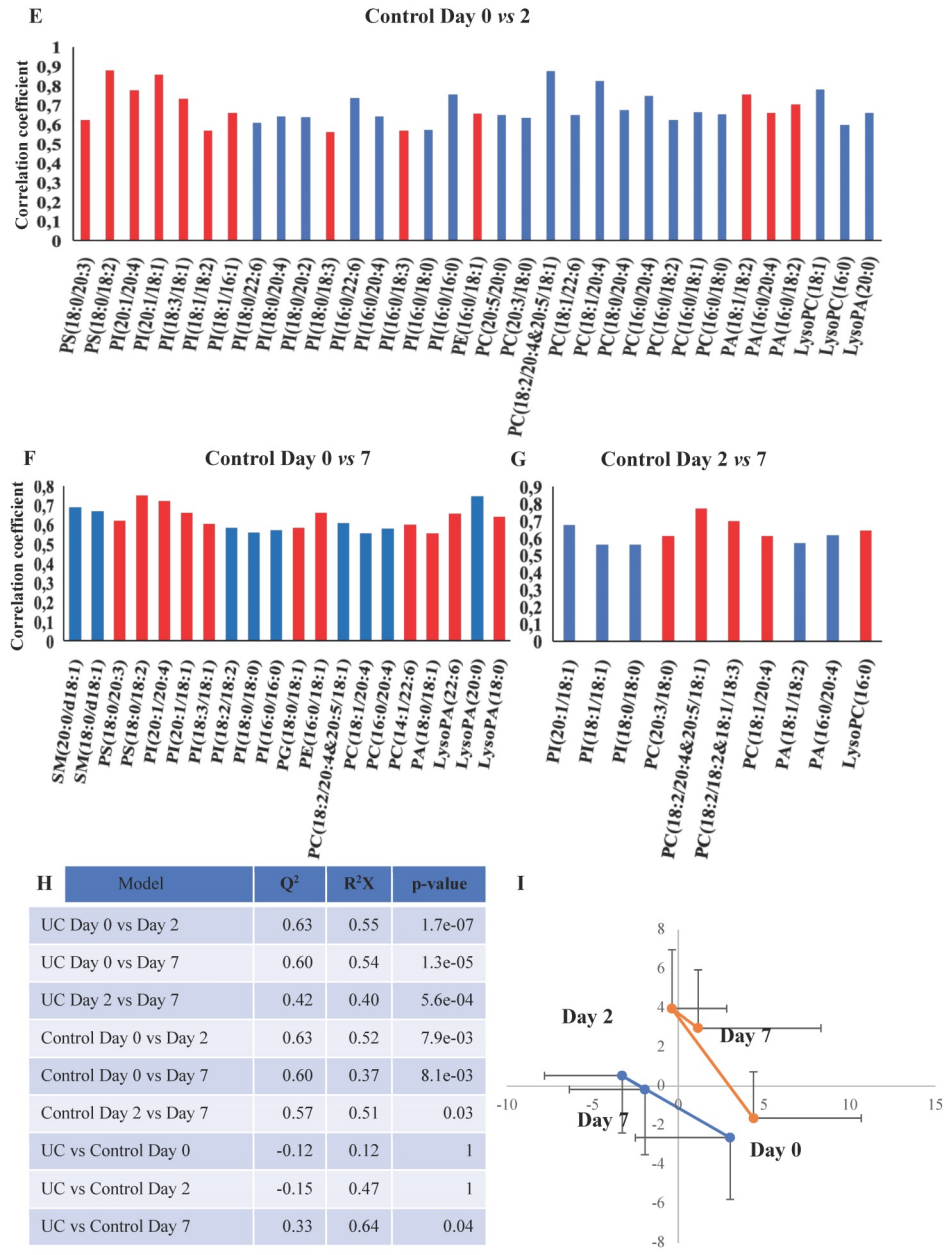
** OPLS-DA models based on phospholipids.** A-G: Phospholipids with correlation coefficients above 0.41 (UC) and 0.60 (controls) indicating significant differential power in the corresponding valid OPLS-DA models. Red, increased levels. Blue, decreased levels. H: Q^2^, cross-validation parameter indicating the predictability of the model. R^2^X, the fraction of the variation of the variables explained by the model. P-values (significant below 0.05) are based on the CV-ANOVA. I: Metabolic trajectory generated from the average of PCA scores of controls (orange color) and UC (blue) during wound healing. Lysophosphatidic acid (LPA), lysophosphatidylcholine (LPC), phosphatidic acid (PA), phosphatidylcholine (PC), phosphatidylethanolamine (PE), phosphatidylglycerol (PG), phosphatidylinositol (PI), phosphatidylserine.

OPLS-DA models based on phospholipids were valid at all three time points during wound healing in both UC and controls, and corresponding significant changes were identified in several phospholipids with both increased and decreased levels (Figure 5A-F, H). Especially UC had a high number of significantly (p<0.05) affected phospholipids, and the difference between UC and controls reached significance at day seven (Figure 5G), where levels of various species of phosphatidylserine (PS), phosphatidylinositol (PI), PG, PC, PA as well as LPA were significantly lower in patients with UC. The metabolic trajectory of this process is illustrated in Figure 5I, where patients with UC and controls had a similar starting point, but subsequently the paths diverged towards day two, and the return (i.e., day seven during wound healing) was notably different.

**Figure 6:**
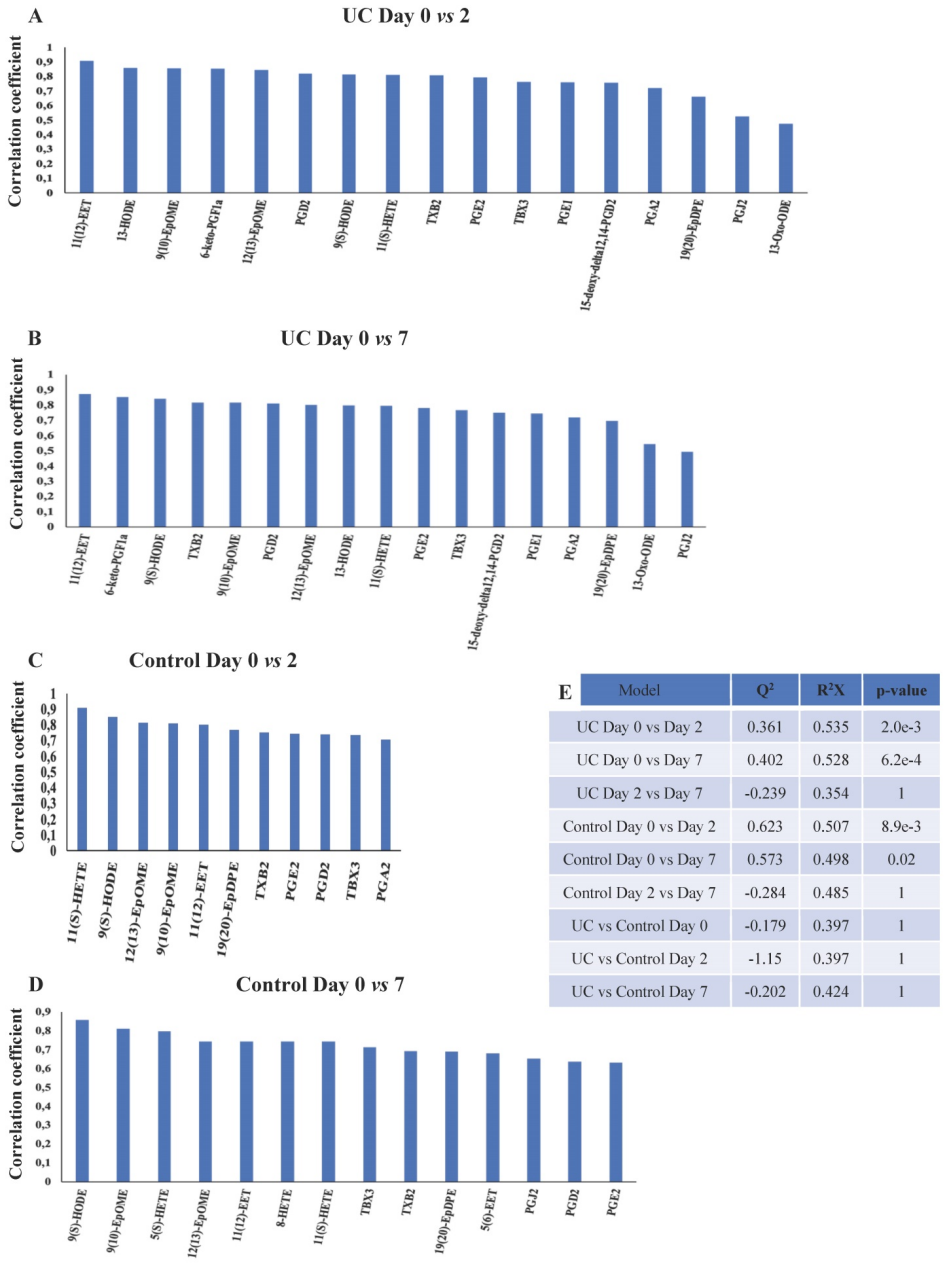
** OPLS-DA models based on eicosanoids.** A-D: Eicosanoids with correlation coefficients above 0.41 (UC) and 0.60 (controls) indicating significant differential power in the corresponding valid OPLS-DA models. All measured eicosanoids were found at lower levels at day two and seven. E: Q^2^, cross-validation parameter indicating the predictability of the model. R^2^X, the fraction of the variation of the variables explained by the model. P-values (significant below 0.05) are based on the CV-ANOVA. Eicosatetraenoic acid (ETE), epoxy-docosapentaenoic acid (EpDPE), epoxyeicosatrienoic acids (EET), epoxy-octadecenoic acid (EpOME), hydroxyeicosapentanenoic acid (HEPE), hydroxyeicosatetraenoic acids (HETE), hydroxy-octadecadienoic acid (HODE), hydroperoxy-eicosapentanenoic acid (HpEPE), octadecadienoic acid (ODE), prostaglandin (PG), thromboxane (TX), ulcerative colitis (UC).

OPLS-DA models for the eicosanoids were valid when day two and seven were compared to day zero for both UC and controls (Figure 6A-E). Again, UC had a higher number of significantly (p<0.05) affected eicosanoids as compared to controls, but no significant differences were found between UC and controls. Thus, significant temporal changes in the levels of eicosanoids were identified during wounding and wound healing, but the metabolic trajectory did not differ significantly between UC and controls.

**Figure 7:**
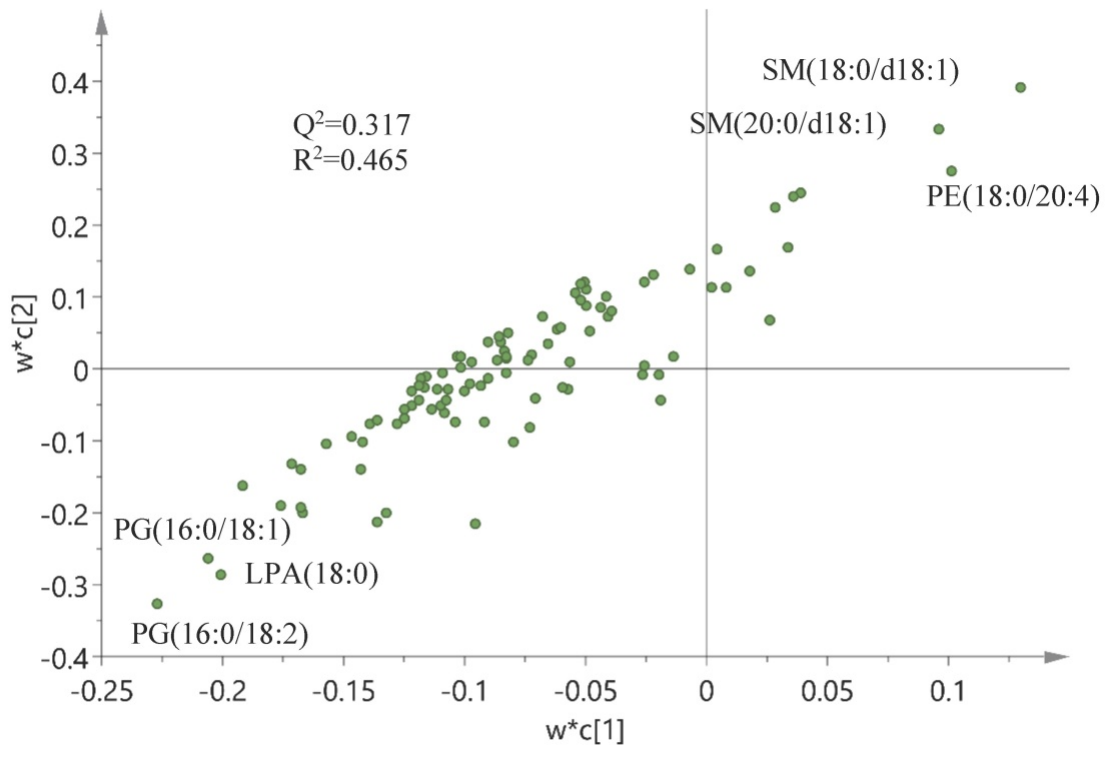
**Correlation between the wound score and phospholipids.** Correlation analysis between wound scores (X-matrix, w*c^1^) and phospholipids (Y-matrix, w*c^2^) based on an OPLS-DA model and corresponding loadings. Q^2^, cross-validation parameter indicating the predictability of the model in relation to its statistical validity. R^2^, the fraction of the variation of the variables explained by the model. Lysophosphatidic acid (LPA), phosphatidylethanolamine (PE), phosphatidylglycerol (PG), sphingomyelin (SM).

Finally, a correlation analysis based on an OPLS-DA model and corresponding loadings was performed between wound scores (X-matrix) and phospholipids (Y-matrix) (Figure 7). As seen in this figure, wound scores were highly correlated with the phospholipid profile - especially with changes in sphingomyelin (SM) and phosphatidylethanolamine (PE) species - which were positively correlated with the wound scores, whereas levels of PG and LPA were negatively correlated. A similar model was built between wound scores and eicosanoids, but this model turned out to be invalid, i.e., failed cross-validation and the permutation test, and hence no correlation could be identified between the wound score and eicosanoids (data not shown).

## Discussion

The human *in vivo* wound healing assay demonstrated that patients with even quiescent UC have an increased inflammatory response to intestinal wounding. This is in alignment with recently published data where inflammatory markers in an *in vivo* injury model showed rapid engagement of innate cytokines like interleukin (IL)- 1a/b and adaptive IL-17A along with recruitment of neutrophils and innate lymphoid cells in quiescent UC 23, and with a correlation between the histological grade of inflammatory infiltrate and wound scores. This UC-specific pattern of disrupted intestinal wounding and wound healing correlated in the current study with distinctive changes in the mucosal lipidome. Thus, both univariate, multivariate, and correlation analyses characterized the control group with only few and temporal changes contrasting the more profound and sustained chances in UC.

Previous studies have uncovered significant decreased amounts of LPC and PC in the colonic mucus of patients with UC 25-27, and human lipidomic studies on serum/plasma 28-30 as well as on colonic biopsies 31 have identified extensive alterations in the lipid composition with changed LPC and PC species. The low levels of LPC, and especially PC as seen in UC, might simply be due to an insufficient re-establishment of the mucus layer post-wounding, but might also be directly causative as different species and isomers of both LPC and PC act as pro- or anti-inflammatory stimuli 32. A cell culture-based study suggests that PC inhibits the inflammatory response caused by tumor necrosis factor (TNF)-α through changes in the properties if the cell membrane, thus interfering with the signal transduction 33. Human functional studies are, however, scarce, but a phase III placebo-controlled trial of a modified release of PC has shown to significantly alleviate disease activity in mesalazine-refractory UC 16, Whether this effect is a consequence of the anti-inflammatory properties; improvement of the mucus barrier, or both is, however, still not clarified.

A similar therapeutic effect has been achieved with local LPA in experimental colitis models by modulating intestinal epithelial migration, proliferation, and survival 17,34. Oral application of LPA mitigates colonic wound closure and inhibits TNF-mediated intestinal barrier defects in mice 35,36. No functional or interventional human studies are currently available, but in this study, LPA species are found at significant lower levels in UC at day seven (Figure 5G, Table S1), due to an aberrant inability to increase these LPA species as a response to wounding. LPA is produced through different pathways 37; one involves the hydrolysis of the membrane derived PA, whereas another requires the elimination of the choline moiety from LPC and ultimately PC. Thus, the relatively low level of LPA in UC may be the consequence of transient or sustained suppressed levels of PA and PC, as demonstrated in the present study (Figure 5G), and might be part of the pathophysiology of compromised wound healing in UC.

Significantly suppressed levels of PS species are seen in UC at day seven compared to controls (Figure 5G). Along with its metabolically related membrane phospholipid PE, PS act as an essential metabolite in a balanced initial phase of wound healing, i.e., coagulation 38 and inflammation 39, a balance that seems to be off in UC wound healing as documented in the present study as well as in biopsy material from patients with flaring UC 31.

The majority of PG species remain unaffected by wounding in controls (Figure 5D-F, Table S1), which contrasts the findings in UC, where the PG level was significantly reduced compared to control (Figure 5G). Upon wounding, PG acts as a regulator of differentiation 40, and PG liposome treatment in murine wound models accelerates wound healing 41. A somewhat similar pattern is observed with PI where most affected PI species are significantly decreased after wounding in UC (Figure 5G). Systemic or local treatment with PI inhibits T-cell proliferation and differentiation and ameliorates intestinal inflammation in a 2,4,6-trinitrobenzenesulfonic acid (TNBS)-induced colitis model 42,43. Thus, these pronounced differences in PG and PI between controls and UC might explain the observed delay in wound healing observed in the present study. No human studies currently exist, but future therapies with supplementation of PG and PI could accelerate wound healing in UC by promotion of differentiation and anti-inflammatory effects.

Sphingomyelin, which is the most abundant sphingolipid and an important structural component of the cell membrane, was found in reduced levels, which correlated with high wound scores (Figure 7). Unfortunately, SM does not seem to offer an opportunity for therapeutic intervention, as it also has proinflammatory capabilities 44.

The majority of eicosanoids measured were significantly decreased at day two and/or seven in both UC and controls (Figure 3B and Figure 6A-D), but no differences could be found between day two and seven in UC or controls, or between UC and controls at any given time point (Figure 6E). The remaining eicosanoids were either unaffected or unmeasurable at several time points due to very low concentrations (Table S2).

Only few eicosanoids are well-described in relation to mucosal wound healing, and only so in experimental animal models. As previously described, PGE_2_ promotes early wound repair by inducing WAE cells in mice 10,11, whereas a subsequent increase in PGD_2_ and its metabolites function as a part of the anti-inflammatory response 45. This does, however, not seem to apply to humans, as we find PGE_2_ and PGD_2_, including its metabolites, i.e., PGJ_2_, and 15-deoxy-Δ12,14-PGD_2_, to be continuously reduced during regeneration, especially in UC (Figure 6A, B). These findings are novel and detrimental to what is known from experimental animal models, which stress the importance and need for human experiments, and obviously merits further analyses and functional tests in future human studies. However, it could be hypothesized that this might represent a point of therapeutic intervention, as animal models suggest that PGD_2_ metabolites ameliorates inflammation 46. Similarly, the sustained reduction of PGE_1_, 13-HODE, and 13-oxo-ODE in UC, which are all anti-inflammatory eicosanoids and improve the microcirculation 47, may be substituted, as animal 48 and human 49 studies have shown increased wound healing times with the infusion of PGE_1_.

The disease specific changes found in lipid abundance could be driven by differences in mucosal cell type composition during wounding and regeneration or by changes in the mucosa-near microbiome and cellular lipid metabolism. Accumulating evidence suggests a microbe-host-lipid co-metabolism 50, which seems especially evident for the eicosanoids 51. Our previous data 23 indicates that the mucosal niche microbiota are changed during colonic mucosal injury and closely resembles the changes seen in active UC in terms of reduced α-diversity, bacterial composition, and bacterial load. These changes are similar in both patients with UC and healthy controls, which might explain why the majority of eicosanoids are reduced in the current study, and equally so in both UC and controls. However, this needs to be elucidated further via functional assays, just as the proposed potential therapeutic targets need to be tested and validated in interventional studies. Moreover, adjunctive comprehensive analyses of the wound niche microbiome, the transcriptome and proteome along with further lipidomic analyses could add significantly insight to the assessment of the wound healing model. Especially the role of specialized pro-resolving lipid mediators derived from polyunsaturated fatty acids like docosahexaenoic acid and eicosapentaenoic acid, which recently have been linked to protective actions in intestinal inflammation and to promote wound healing 14, might be of significant interest in future lipidomic studies.

It is important to note that the applied wound healing assay does not mimic the actual inflammation during regular flares of UC, but it is considered a tool to investigate the initial response to mucosal injury and subsequent mucosal healing process. Finally, no age-match was made between patients with UC and healthy control, nor were any restrictions imposed in terms of medication except that it had to be stable for at least 3 months. The latter needs to be kept in mind when interpreting the results, as the majority of patients with UC received maintenance treatment with 5-aminosalicylic acid, which may activate peroxisome proliferator-activated receptors with multiple effects on lipid metabolism, inflammatory processes, and cell proliferation 52.

## Conclusions

In summary, 1) this study describes for the first time the kinetics of disrupted metabolic pathways in the wound healing process in the colon mucosa among patients with quiescent UC compared to healthy individuals. Thus, the analyses identify a persistent inflammatory response and delayed epithelial restitution during wound healing in UC with underlying UC-specific perturbations in lipid mediator engagement. Moreover, 2) the lipidomic characterization presented identifies significant temporal changes of several phospholipids and eicosanoids with significant differences to what is found in comparable animal models. Ultimately, 3) these results challenge our traditional treatment approach of IBD and may guide future medical treatment regimens. Therapeutic interventions ameliorating the low levels of especially LPC, PC, LPA PG, PI, PGD_2_, and PGE_1_ in inflamed colonic tissue may represent a novel potential therapeutic avenue for recovering flaring UC and subsequent epithelial healing, which, however, requires future functional and interventional human studies. In this way lipidomics may provide useful novel insights into specific disease phenotypes, potentially leading towards improved clinical practice, which will enable the development of personalized medicine in a wide range of diseases, including UC.

## Supplementary Material

Supplementary figures and tables, information.

## Figures and Tables

**Table 1:** All patients included with ulcerative colitis (UC) were in deep remission (endoscopic Mayo score 0 and histological normal colonic mucosa, Geboes score < 0.1), and were categorized in accordance with previous extent of the disease. E1: proctitis, E2: left-sided colitis, E3: extensive colitis. EIM: extraintestinal manifestation.

Table 1	Cohort 1		Cohort 2	
Characteristics	Ulcerative colitis n = 8	Healthy controls n = 8	Ulcerative colitis n = 21	Healthy controls n = 9
Gender (male/female)	4/4	4/4	9/12	4/5
Age, years (mean, range)	52 (33-67)	24 (22-26)	48 (19-73)	47 (23-56)
Age at diagnosis (≤25/> 25 years)	0/8	na	8/13	na
Years with disease (≤10/> 10 years)	7/1	na	12/9	na
Extension (E1, E2 or E3)	2/6/0	na	5/10/6	na
Smoking/former/non-smoking	0/0/8	0/0/8	0/6/15	0/0/9
EIM (present/not present)	0/8	na	3/18	na
Previous glucocorticoid (independent/dependent)	8/0	na	14/7	na
Current daily medication, n Systemic 5-aminosalicylic acid (1.6-3.2 g)	5	na	18	na
Current topical 5-aminosalicylic acid (1 g)	0	na	1	na
Current azathioprine (100-150 mg)	0	na	4	na
Previous infliximab (5 mg/kg)	0	na	2	na
None of the above medications	3	8	1	9

## Abbreviations

BHT: butylated hydroxytoluene
CRC: colorectal cancer
CV-ANOVA: analysis of variance of the cross-validated residuals
EET: epoxyeicosatrienoic acids
EpDPE: epoxy-docosapentaenoic acid
EpOME: epoxy-octadecenoic acid
ESI: electrospray ionization
ETE: eicosatetraenoic acid
FDR: false discovery rate
HEPE: hydroxyeicosapentanenoic acid
HETE: hydroxyeicosatetraenoic acids
HODE: hydroxy-octadecadienoic acid
HpEPE: hydroperoxy-eicosapentanenoic acid
IBD: inflammatory bowel disease
IQR: interquartile range
LPA: lysophosphatidic acid
LPC: lysophosphatidylcholine
LPE: lysophosphatidylethanolamine
LC-MS: liquid chromatography mass spectrometry
LT: leukotriene
MRM: multiple reaction-monitoring
ODE: octadecadienoic acid
OPLS-DA: orthogonal projection to latent structure-discriminant analysis
PA: phosphatidic acid
PC: phosphatidylcholine
PCA: principal component analysis
PE: phosphatidylethanolamine
PG: phosphatidylglycerol
PGA/D/E/J: prostaglandin A/D/E/J
PI: phosphatidylinositol
PS: phosphatidylserine
SM: sphingomyelin
TNBS: trinitrobenzenesulfonic acid
TNF: tumor necrosis factor
TX: thromboxane
UC: ulcerative colitis
UV: unit variance
UPLC-MS: ultraperformance liquid chromatography-mass spectrometry
WAE: wound associated epithelial
