# Supplementary material for: Lipidomic Trajectories Characterize Delayed Mucosal Wound Healing in Quiescent Ulcerative Colitis and Identify Potential Novel Therapeutic Targets
Source: Int J Biol Sci. 2022 Feb 14;18(5):1813–28. doi: 10.7150/ijbs.67112 (PMC8935238; doi:10.7150/ijbs.67112)
Supplement: Supplementary file 1 — Supplementary figures and tables, information. [file ijbsv18p1813s1.pdf]

Supplementary material

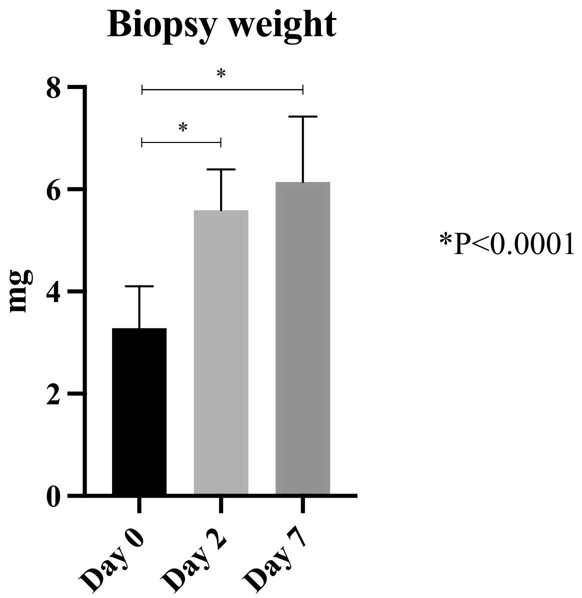

Figure 1. Biopsy wet weight from Cohort 2
Similar data not available from Cohort 1.
Median and interquartile range

Phospholipids	UC						Controls					
	Day 0		Day 2		Day 7		Day 0		Day 2		Day 7	
	Median	IQR	Median	IQR	Median	IQR	Median	IQR	Median	IQR	Median	IQR
**20:1 LPE**	52,0	44,1	0,0	0,0	0,0	0,0	40,3	25,0	0,0	0,0	24,1	2,3
**16:0 LPC**	36,3	20,9	**31,5***	**16,1**	41,0	19,2	40,1	28,1	29,6	10,1	49,2	19,2
**18:2 LPC**	12,9	7,6	**8,1***	**6,5**	8,0	2,6	10,6	3,6	9,8	9,8	7,6	5,3
**18:1 LPC**	25,7	16,0	**12,8***	**9,6**	**15,6***	**8,3**	27,2	14,6	13,9	22,1	16,8	11,6
**18:0 LPC**	16,9	9,2	**10,9***	**3,1**	15,2	6,6	18,8	7,0	10,6	4,9	15,1	7,3
**20:4 LPC**	0,0	0,0	6,9	0,3	0,0	0,0	0,0	0,0	0,0	0,0	4,4	0,0
**Total LPC**	93,1	52,8	**61,0***	**29,3**	81,0	37,8	92,3	57,7	73,6	39,2	90,2	38,9
**16:0/18:1 PE**	15,2	5,7	**11,3***	**3,5**	9,8	3,0	16,7	0,0	10,7	2,0	8,6	3,1
**18:2/16:0 PE**	14,0	5,0	11,4	1,8	7,9	0,7	18,2	0,0	9,8	2,5	8,1	1,2
**18:1/18:2 PE**	18,8	11,8	**16,5***	**5,3**	17,0	5,9	17,9	5,5	15,8	4,1	18,8	4,5
**18:0/18:1 PE**	23,8	9,3	**14,1***	**3,1**	**13,1***	**3,1**	18,6	9,8	14,5	2,6	12,4	3,1
**18:0/20:4 PE**	34,1	13,4	**24,4***	**10,5**	**22,0***	**6,2**	20,0	12,2	21,3	5,4	20,7	4,2
**Total PE**	65,0	44,7	**54,2***	**48,0**	54,2	22,7	53,7	37,9	64,2	17,6	67,1	18,7
**22:6 LPA**	0,9	0,1	0,0	0,0	0,6	0,8	0,9	0,0	0,7	0,2	0,8	0,2
**18:0 LPA**	1,2	0,6	0,9	0,9	0,7	0,4	1,1	0,0	0,8	0,3	0,9	0,3
**20:0 LPA**	5,0	3,0	**2,9***	**1,7**	**3,1***	**1,8**	5,1	1,7	**3,1***	**1,2**	3,2	1,5
**Total LPA**	6,1	2,2	2,88*	2,2	**3,5***	**1,9**	5,9	1,7	3,8	2,1	4,7	2,6
**16:0/18:2 PA**	0,7	0,5	**1,2***	**0,8**	0,6	1,7	0,8	1,0	2,1	2,0	1,6	2,4
**18:0/16:0 PA**	1,0	0,5	0,7	0,5	0,4	0,4	0,8	0,3	0,3	0,1	0,4	0,6
**16:0/20:4 PA**	0,5	0,3	0,6	0,5	0,4	1,1	0,6	0,3	0,9	0,8	0,7	0,9
**18:1/18:2 PA**	0,5	0,5	0,7	0,4	1,3	1,1	0,6	0,1	0,8	1,0	0,7	1,3
**18:0/18:1 PA**	7,1	3,9	**3,9***	**3,0**	4,5	4,9	7,2	4,3	11,0	11,5	16,7	11,9
**18:0/18:0 PA**	20,0	9,9	**12,3***	**4,5**	12,6	9,3	16,5	7,1	14,8	10,4	21,8	12,9
**18:0/20:4 PA**	456,3	170,8	**193,8***	**106,1**	**228,4***	**150,8**	357,3	216,4	240,7	135,4	236,9	192,3
**22:6/22:6 PA**	13,7	10,3	**8,9***	**4,1**	9,5	11,4	11,4	6,1	10,4	4,5	13,3	6,6
**Total PA**	500,2	191,0	**224,1***	**107,7**	**276,7***	**180,5**	390,2	213,7	273,2	166,4	283,9	223,6
**16:0/18:2 PG**	0,2	0,1	0,2	0,2	**0,3****	**0,2**	0,4	0,4	0,4	0,2	0,7	0,8
**16:1/18:1 PG**	33,3	12,5	**23,4***	**12,7**	24,3	9,9	31,2	19,9	31,3	8,7	32,3	19,2
**16:0/18:1 PG**	2,9	1,2	**2,0***	**1,0**	2,0	1,2	3,3	1,7	3,4	1,0	3,3	2,4
**16:1/20:4 PG**	3,7	1,6	**2,1***	**1,3**	**2,2***	**0,9**	3,4	2,0	2,7	0,6	3,0	1,6
**18:2/18:2 PG**	10,1	4,0	**7,0***	**4,7**	8,9	4,1	9,5	5,3	9,9	5,9	12,3	6,2
**18:0/18:1 PG**	1,3	0,4	1,3	0,5	1,6	0,9	2,0	1,5	2,5	2,3	3,4	4,6
**Total PG**	52,3	19,4	**37,3***	**19,8**	37,3	16,2	51,6	28,7	50,3	17,1	55,7	33,8

**18:1/18:1 PS**	11,7	3,7	**10,1***	**4,4**	8,4	5,4	14,4	5,0	14,1	5,9	15,4	10,6
**18:0/18:2 PS**	6,5	1,7	**8,0***	**4,0**	6,5	6,2	6,8	2,0	**13,1***	**7,6**	10,1	5,1
**18:0/18:1 PS**	112,1	42,8	**94,4***	**41,4**	85,4	85,5	132,1	48,0	157,8	76,7	158,0	83,0
**18:0/18:0 PS**	162,7	54,1	**137,9***	**30,4**	124,8	54,5	158,5	69,1	148,9	38,6	187,1	74,4
**18:0/20:4 PS**	10,0	3,4	9,8	3,3	9,7	6,3	9,4	5,1	12,1	4,1	9,8	8,3
**18:0/20:3 PS**	2,7	0,7	2,1	0,6	2,2	1,6	2,3	1,2	2,6	1,9	2,7	2,2
**20:0/18:1 PS**	3,8	1,5	**2,8***	**1,3**	2,4	1,9	4,8	2,0	4,2	2,7	5,4	1,6
**18:0/22:6 PS**	5,9	3,5	6,5	2,7	7,7	5,1	5,6	2,7	7,7	3,5	7,6	2,8
**Total PS**	322,3	90,6	**270,0***	**81,2**	245,6	150,5	358,3	113,3	359,6	130,7	423,5	166,3
**16:0/16:1 PI**	2,0	1,8	1,9	0,7	2,0	0,9	1,9	1,1	2,3	0,8	2,6	2,4
**16:0/16:0 PI**	3,5	1,7	**2,1***	**1,1**	2,6	1,7	4,0	1,5	**2,6***	**1,1**	2,9	2,2
**16:1/18:2 PI**	0,3	0,2	0,3	0,1	0,3	0,2	0,4	0,3	0,3	0,1	0,3	0,2
**16:0/18:3 PI**	0,6	0,5	0,5	0,4	0,5	0,3	0,4	0,3	0,5	0,3	0,5	0,4
**16:0/18:2 PI**	14,6	8,1	**12,0**	**6,2**	13,9	9,9	15,9	5,9	14,5	3,9	16,3	7,2
**18:1/16:1 PI**	1,2	0,8	**1,4***	**0,6**	1,0	0,6	1,0	0,6	1,6	1,3	1,6	1,4
**16:0/18:1 PI**	19,2	4,8	**14,0***	**6,3**	14,2	7,6	22,5	7,7	16,5	6,4	17,3	11,7
**18:0/16:1 PI**	12,9	5,5	14,1	6,1	**15,0***	**7,3**	12,0	3,1	13,8	5,6	14,9	3,7
**16:0/18:0 PI**	17,0	5,7	**12,8***	**4,5**	12,9	4,9	17,2	9,1	**13,7***	**2,7**	13,1	9,7
**16:0/20:5 PI**	0,7	0,3	**0,55***	**0,5**	0,7	0,6	0,9	0,8	0,6	0,5	0,9	0,5
**18:2/18:2 PI**	0,7	0,4	**0,5***	**0,3**	0,5	0,4	0,8	0,6	0,6	0,3	0,4	0,4
**18:3/18:1 PI**	0,4	0,4	0,2	0,3	0,2	0,1	0,3	0,0	0,3	0,3	0,2	0,2
**16:0/20:4 PI**	10,5	4,4	**7,6***	**2,8**	8,0	4,1	9,9	4,4	**7,7***	**1,2**	8,6	5,6
**18:1/18:2 PI**	6,0	2,8	**6,8***	**1,9**	5,6	3,3	5,7	2,7	8,5	2,5	6,8	4,0
**16:0/20:3 PI & 20:3/16:0 PI**	2,6	1,6	**2,0***	**0,9**	2,3	1,5	3,1	0,8	2,1	0,4	2,9	1,8
**18:0/18:3 PI**	1,3	1,8	1,3	0,9	1,1	1,0	1,3	0,5	1,7	1,0	1,4	0,9
**18:0/18:2 PI**	21,5	12,5	18,9	7,8	17,7	11,3	26,9	9,2	24,8	6,7	23,3	10,4
**18:1/18:1 PI**	11,3	5,0	12,4	4,2	10,3	4,5	12,7	8,7	15,7	7,5	12,6	10,4
**16:0/20:2 PI**	0,6	0,3	**0,4***	**0,2**	**0,4***	**0,2**	0,6	0,3	0,3	0,3	0,4	0,2
**18:0/18:1 PI**	21,2	10,3	19,2	7,9	16,9	9,4	28,9	11,7	24,8	11,1	23,0	16,8
**18:0/18:0 PI**	30,1	11,2	**24,9***	**6,8**	20,1	8,0	29,2	14,3	25,9	7,3	22,6	10,2
**18:1/20:5 PI**	0,4	0,2	0,3	0,2	0,3	0,2	0,5	0,4	0,4	0,2	0,4	0,2
**16:0/22:6 PI**	2,5	1,4	**1,4***	**1,0**	2,0	1,5	2,9	1,5	**1,4***	**0,9**	2,6	1,9
**18:1/20:4 PI**	7,2	3,2	**6,1***	**2,1**	6,2	3,6	6,7	2,2	7,0	1,3	6,6	4,7
**18:0/20:5 PI**	2,1	2,0	2,3	1,5	2,5	1,7	3,1	2,0	2,8	2,0	3,6	2,2
**18:0/20:4 PI**	55,9	23,3	**37,9***	**6,8**	43,0	25,9	58,8	16,7	**41,3***	**9,9**	39,4	25,1
**18:1/20:3 PI**	0,7	0,6	0,9	0,5	0,8	0,5	1,2	0,6	1,1	0,6	1,3	0,8

**18:0/20:3 PI**	7,6	3,0	**5,2***	**2,2**	6,2	3,9	8,4	3,0	6,6	2,3	6,7	5,0
**18:0/20:2 PI**	11,4	5,4	**9,1***	**3,7**	8,6	5,1	11,4	7,0	**7,8***	**2,0**	9,4	5,5
**20:1/18:1 PI**	0,3	0,1	**0,4***	**0,2**	0,3	0,2	0,3	0,1	0,5	0,3	0,4	0,4
**18:0/22:6 PI**	2,7	1,2	**2,2***	**1,3**	2,6	1,8	3,6	1,4	**2,2***	**1,1**	2,5	2,1
**20:1/20:4 PI**	0,2	0,0	0,2	0,1	0,2	0,1	0,2	0,0	0,2	0,0	0,2	0,2
**20:0/20:4 PI**	0,3	0,2	**0,28***	**0,1**	0,3	0,1	0,3	0,4	0,3	0,1	0,4	0,3
**Total PI**	252,4	88,7	**216,6***	**43,8**	211,4	96,5	277,5	117,7	264,0	55,5	272,4	123,6
**16:0/14:0 PC**	61,5	21,2	56,3	18,6	71,3	17,6	52,8	13,6	58,0	24,4	76,1	40,8
**16:0/16:1 PC & 14:0/18:1 PC**	117,9	52,7	111,3	54,6	151,9	52,9	98,5	58,9	115,8	61,5	161,9	119,6
**16:0/16:0 PC**	430,4	220,9	**317,6***	**114,4**	416,1	151,3	332,4	109,7	308,7	87,6	388,8	213,8
**16:0/18:2 PC**	1047,3	426,7	**852,0***	**391,5**	977,5	408,6	1056,1	282,0	**847,8***	**177,4**	976,9	386,7
**16:0/18:1 PC**	2056,9	912,3	**1505,7***	**606,7**	**1647,5***	**533,2**	2039,9	952,6	**1495,8***	**451,9**	1884,1	1107,2
**16:0/18:0 PC**	1302,8	560,7	**977,6***	**397,2**	1104,1	331,8	1315,1	650,3	**967,4***	**317,4**	1228,7	698,3
**18:0/16:0 PC**	65,7	32,8	**53,1***	**19,9**	60,1	16,3	56,2	9,5	51,3	19,0	55,9	33,6
**14:1/22:6 PC**	4,8	2,7	3,3	1,0	3,2	1,0	4,1	2,0	3,7	1,3	4,1	4,4
**16:0/20:5 PC**	27,0	18,9	**20,7***	**17,8**	27,7	22,5	34,2	15,5	**16,5***	**15,0**	27,1	16,8
**18:2/18:2 PC & 18:1/18:3 PC**	72,5	33,7	**53,7***	**53,7**	65,6	40,0	83,7	33,2	**58,5***	**34,9**	94,3	53,3
**16:0/20:4 PC**	506,5	291,8	**296,5***	**100,1**	**286,0***	**137,4**	433,5	150,0	**251,6***	**65,7**	302,0	131,6
**18:0/18:1 PC**	375,3	167,5	**312,0***	**106,2**	**297,3***	**83,7**	381,9	160,5	292,2	81,1	345,3	161,4
**20:0/16:1 PC**	64,4	34,1	**54,0***	**14,9**	**47,3***	**12,8**	68,0	24,3	51,1	29,0	49,6	27,7
**18:0/18:0 PC**	296,3	120,6	**241,6***	**80,1**	**231,0***	**65,8**	297,9	126,5	228,9	65,1	272,1	126,3
**18:2/20:4 PC & 20:5/18:1 PC**	40,5	14,6	**19,9***	**18,6**	26,6	14,0	41,6	10,4	**23,1***	**16,0**	34,6	12,5
**18:1/20:4 PC**	176,1	102,9	**86,4***	**52,6**	**102,3***	**48,6**	165,7	28,7	**95,6***	**56,3**	126,7	44,8
**18:0/20:4 PC**	295,8	176,5	**185,5***	**88,2**	**207,5***	**85,1**	259,0	103,3	**166,7***	**58,1**	182,7	115,1
**20:3/18:0 PC**	21,9	11,2	**14,6***	**7,9**	17,1	8,4	23,1	9,1	**14,4***	**8,2**	22,9	9,9
**18:1/22:6 PC**	15,5	9,2	**10,6***	**7,2**	11,2	7,3	19,1	12,8	**9,5***	**8,3**	13,5	11,1
**20:5/20:0 PC**	19,5	12,8	**12,7***	**4,9**	14,7	5,7	16,1	5,2	**10,9***	**4,3**	14,9	5,6
**22:0/18:3 PC**		0,0	0,0	0,0	1,8	0,0	0,0	0,0	2,7	0,9	1,8	0,0
Total PC	7048,1	3066,5	**5380,8***	**1969,0**	5741,4	1715,4	6617,4	2407,7	**4862,2***	**1301,8**	6158,0	3208,9
**14:0/d18:1 SM**	18,5	10,5	19,8	4,8	23,5	8,5	16,8	6,1	21,6	9,3	25,7	13,9
16:0/d18:1 SM	624,4	272,9	**534,4***	**159,3**	617,3	197,8	620,0	224,5	567,2	202,4	668,8	451,1
16:0/d18:0 SM	348,1	152,5	**301,5***	**84,5**	339,2	114,0	353,6	136,5	316,7	120,2	375,1	264,5
18:0/d18:1 SM	69,2	53,7	**51,5***	**20,5**	46,7*	14,9	74,9	29,4	54,7	23,1	51,1	20,9
20:0/d18:1 SM	31,7	20,9	**22,1***	**8,6**	18,9*	5,3	33,1	11,1	**24,9***	**10,9**	19,8	8,2
**22:0/d18:1 SM**	67,8	38,9	**57,4***	**15,1**	**49,8***	**14,8**	69,8	23,7	52,0	27,4	51,3	28,0

**24:1/d18:1 SM**	201,6	109,0	**167,8***	**51,3**	162,6	54,3	189,7	67,6	158,7	75,4	165,7	108,2
**24:0/d18:1 SM**	73,5	34,1	**56,1***	**20,5**	58,2	18,3	72,0	31,9	53,3	40,6	53,8	40,0
**Total SM**	1421,4	731,7	**1260,3***	**300,5**	1304,9	401,4	1419,9	501,7	1277,1	498,8	1372,6	948,4

Table 1. Measured phospholipids (pmol/mg) during wound healing in ulcerative colitis and controls. Interquartile range (IQR), Lysophosphatidic acid (LPA), lysophosphatidylcholine (LPC), lysophosphatidylethanolamine (LPE), phosphatidic acid (PA), phosphatidylcholine (PC), phosphatidylethanolamine (PE), phosphatidylglycerol (PG), phosphatidylinositol (PI), phosphatidylserine (PS), sphingomyelin (SM), ulcerative colitis (UC).
*: comparing day two or seven with day zero for UC or controls and False Discovery Rate $p<0.05$.
**: comparing UC and controls at day 7 and False Discovery Rate $p<0.05$.

	UC						Controls					
**Eicosanoids**	**Day 0**		**Day 2**		**Day 7**		**Day 0**		**Day 2**		**Day 7**	
	**Median**	**IQR**	**Median**	**IQR**	**Median**	**IQR**	**Median**	**IQR**	**Median**	**IQR**	**Median**	**IQR**
**LTE4**	020	0,150	0,003	0,027	0,022	0,087	0,000	0,178	0,000	0,108	0,001	0,149
**TXB2**	0,361	0,276	**0,006***	**0,012**	**0,005***	**0,009**	0,449	0,343	**0,006***	**0,025**	**0,006***	**0,012**
**6-keto-PGF1a**	1,429	2,762	**0,012***	**0,018**	**0,002***	**0,022**	0,957	4,338	**0,005***	**0,026**	**0,010***	**0,032**
**TBX3**	0,183	0,164	**0,002***	**0,010**	**0,002***	**0,009**	0,175	0,200	**0.000***	**0,003**	**0.000***	**0,022**
**PGE1**	1,222	1,527	**0,164***	**0,268**	**0,111***	**0,327**	1,069	2,065	0,000	0,150	0,080	0,367
**13,14-2OH-15-keto-PGD1**	0,000	0,023	0,000	0,023	0,000	0,001	0,000	0,006	0,0,004	0,008	0,000	0,008
**13,14-2OH-15keto-PGD2**	0,196	0,519	**0.00***	**0,044**	**0,000***	**0,237**	0,000	0,335	0,000	0,008	0,000	0,001
**PGD2**	4,524	3,044	**0,103***	**0,192**	**0,174***	**0,352**	5,789	7,246	**0,093***	**0,492**	**0,161***	**0,247**
**PGE2**	3,951	3,152	**0,097***	**0,176**	**0,114***	**0,164**	6,044	5,803	**0.000***	**0,422**	0,122	0,250
**19(20)-EpDPE**	0,248	0,166	**0,020***	**0,075**	**0,019***	**0,058**	0,264	0,251	**0,029***	**0,062**	**0,019***	**0,063**
**LTB4**	0,001	0,011	0,000	0,002	0,000	0,002	0,001	0,003	0,000	0,002	0,000	0,005
**15-deoxy-delta12,14-PGD2**	0,184	0,195	**0,000***	**0,014**	**0,002***	**0,020**	0,205	0,286	**0,009***	**0,020**	0,000	0,019
**PGA2**	0,144	0,166	**0,004***	**0,014**	**0.000***	**0,010**	0,209	0,249	**0,005***	**0,031**	0,004	0,023
**PGJ2**	0,231	0,374	**0,019***	**0,140**	**0.000***	**0,077**	0,347	0,808	0,013	0,077	0,000	0,009
**11(S)-HETE**	0,919	0,963	**0,100***	**0,228**	**0,134***	**0,190**	0,958	0,377	**0,116***	**0,244**	**0,107***	**0,178**
**5(S)-HETE**	0,242	0,341	0,092	0,165	0,175	0,202	0,400	0,352	0,184	0,255	**0,045***	**0,151**
**15(S)-HEPE**	0,000	0,054	0,000	0,006	0,000	0,000	0,000	0,064	0,000	0,001	0,000	0,009
**12(S)-HEPE**	0,002	0,063	0,000	0,040	0,000	0,007	0,000	0,039	0,000	0,034	0,005	0,097
**5(S)-HEPE**	0,010	0,252	0,031	0,135	**0,018***	**0,235**	0,012	0,221	0,019	0,172	0,000	0,059
**12(S)-HETE**	0,016	0,053	0,000	0,020	0,003	0,104	0,000	0,126	0,012	0,116	0,016	0,065
**15-HpEPE**	0,000	1,050	0,500	1,035	0,000	0,534	0,000	1,319	0,250	0,877	0,000	1,067
**12(13)-EpOME**	1,855	1,767	**0,356***	**0,240**	**0,303***	**0,256**	1,392	0,737	**0,424***	**0,508**	**0,419***	**0,502**
**9(10)-EpOME**	3,414	3,116	**0,410***	**0,536**	**0,404***	**0,439**	3,880	1,568	**0,609***	**1,171**	**0,652***	**1,197**
**9(S)-HODE**	3,575	3,191	**0,471***	**0,515**	0,379	0,487	4,317	1,499	**0,378***	**1,473**	**0,524***	**0,918**
**13-HODE**	1,750	1,602	**0,406***	**0,247**	**0,322***	**0,381**	1,526	1,182	0,466	0,477	0,404	0,796
**13-Oxo-ODE**	1,006	0,542	**0,479***	**0,418**	**0,517***	**0,451**	1,279	0,709	**0,618***	**0,738**	**0,603***	**0,607**
**12-oxo-ETE**	0,000	0,079	0,000	0,013	0,000	0,002	0,000	0,000	0,000	0,048	0,000	0,032
**5-oxo-ETE**	0,000	0,993	0,000	0,567	0,000	0,504	0,000	1,192	0,000	0,031	0,066	0,499
**8-HETE**	0,000	0,061	0,000	0,009	0,002	0,020	0,081	0,114	**0.000***	**0,020**	0,000	0,006
**11(12)-EET**	0,885	0,690	**0,101***	**0,181**	**0,106***	**0,182**	1,066	0,501	**0,146***	**0,197**	**0,206***	**0,250**
**5(6)-EET**	0,000	0,019	0,000	0,004	0,000	0,002	0,074	0,119	**0,001***	**0,032**	**0.000***	**0,019**
**14,15-Di-HETE**	0,228	2,588	0,015	0,433	0,092	0,459	2,212	6,533	0,017	0,446	0,134	1,440
**11,12-Di-HETE**	0,013	0,040	**0,003***	**0,018**	**0,002***	**0,010**	0,000	0,021	0,006	0,020	0,003	0,007

Table 2. Measured eicosanoids (pmol/mg) during wound healing in ulcerative colitis and controls. Eicosatetraenoic acid (ETE), epoxy-docosapentaenoic acid (EpDPE), epoxyeicosatrienoic acids (EET), epoxy-octadecenoic acid (EpOME), hydroxyeicosapentanenoic acid (HEPE), hydroxyeicosatetraenoic acids (HETE), hydroxy-octadecadienoic acid (HODE), hydroperoxy-eicosapentanenoic acid (HpEPE), Interquartile range (IQR), leukotriene (LT), octadecadienoic acid (ODE), prostaglandin (PG); thromboxane (TX), ulcerative colitis (UC).
*: comparing day two or seven with day zero for UC or controls and False Discovery Rate $p<0.05$.

Internal standard and chemicals

Phospholipid internal standards are 17:0 PE, 17:1 LPC, 17:1 lysophosphatidylethanolamine (LPE), 17:0 LPA, 17:0 PA, 17:0 PG, 17:0/14:1 PI, 17:0 PS, 17:0 PC, 17:0/d18:1 SM. Internal standards for eicosanoids are 6-keto-PGF1a-d4, PGF2-d4, 5(S)-HETE-d8, 12(S)-HETE-d8 and 9-HODE-d8. These internal standards were purchased from Avanti Polar Lipids Inc. 3, 5-Di-tert-4-butylhydroxytoluene (BHT) were purchased from Supelco, Sigma Aldrich. Methanol (hypergrade LC-MS) and formic acid (LC-MS grade) used were purchased from Merck. Water and ammonium acetate used were of Optima LC-MS grade, purchased from Fisher Scientific.

Quantification analysis

We have previously developed a method to quantify 10 classes of phospholipids [1]: 1) systematically screening all possible lipid combination for each class of phospholipids, 2) Identification of position and structure of the lipids for each class of phospholipids, after which we know the retention time and multiple reaction monitoring (MRM) transitions of the structure of the phospholipids; 3) quantification is now possible using MRM. The quantification analysis was processed manually using MassLynx (Waters) and TargetLynx (Waters), respectively. The concentrations of phospholipid species from classes of PE, LPC, LPE, LPA, PA, PG, PI, and phosphatidylserine (PS) were quantified based on the relative peak area of these phospholipids to the respective internal standards of the same class, cf. Suppl. Table 1. The concentrations of PC and

SM were calculated based on the relative peak area of these phospholipids in the sample to 17:0 PC and 17:0/d18:1 SM in the QC, respectively. The quantification of eicosanoids was calculated based on peak area of the internal standard that was eluted at similar retention time to respective eicosanoids. The concentrations obtained were then normalized by the wet weight of the tissue samples and expressed as pmol/mg.

1. Huang Q, Lei H, Dong M, Tang H, Wang Y. Quantitative analysis of 10 classes of phospholipids by ultrahigh-performance liquid chromatography tandem triple-quadrupole mass spectrometry. Analyst. 2019; 144: 3980–3987.
